# Old German Remedy for the Tooth-Ache

**Published:** 1841

**Authors:** 


### AN OLD GERMAN REMEDY FOR TOOTH-ACHE.

Of the various kinds of nostrums, or quack medicines, that bold or cunning ingenuity has been able to invent, none have been more anxiously seized upon than those which have purported to cure the tooth-ache; and one has no sooner failed or fallen into disrepute, than another has been gotten up. Every year almost, from time immemorial, has produced its scores of remedies, and the loss of confidence in one, has only served to inspire it afresh in the virtues of another; and thus credulity with a liberal hand, has ever been ready to reward those who under the guise of benefactors, have sought only to impose upon the credence of the afflicted or unfortunate.

With most persons afflicted with tooth-ache, any thing is preferable to having the offending organ removed; that is the last remedy. In looking over an old No. of "*the Monthly Magazine*," for *September*, 1798, we came across the following and we do not for a moment doubt that the confidence of Dr. Hirsch in the anti-odontalgic virtues of the *Caccinella*, ridiculous as it may seem, was, as he has here represented it.——BALT. ED.

"In the practice of my profession," says Doctor Frederick Hirsch, Dentist to several German Courts, "I have particularly turned my attention to the cure of the tooth-ache, and I learned from a celebrated German Physician, as well as from the *Journal der Erfindungen*, &c. Journal of Conventions, Theories and Contradictions, in Natural Phylosophy and Physic, No. XIV.

p. 135, that among other insects, the well known lady-bird, *caccinella septempunctata*, possessed a peculiar virtue against the tooth-ache ; I was induced to collect some of these insects ; on repeated trials I found it to exceed my expectations, and I was so happy as to cure several persons speedily and completely with the small insects ; finding myself obliged to repeat the remedy only in the cases of a few female patients. My method of proceeding was as follows : I crushed the insect between my thumb and forefinger, and rubbed it between them until their points grew warm ; with the forefinger and thumb thus prepared, I then rubbed the affected parts of the gum, and the aching tooth ; upon which the pains in every instance, except in the cases mentioned above, completely ceased ; I found likewise, that the medicinal virtue of this insect was so powerful and durable, that my forefinger was capable of remedying the tooth-ache, for some days after, without crushing an insect on it afresh."

